# Development of an Analytical Method for Dibutyl Phthalate Determination Using Surrogate Analyte Approach

**Published:** 2017

**Authors:** Vahid Farzanehfar, Mehrdad Faizi, Nima Naderi, Farzad Kobarfard

**Affiliations:** a *Department of Pharmacology and Toxicology, School of Pharmacy, Shahid Beheshti University of Medical Sciences, Tehran, Iran. *; b *Department of Medicinal Chemistry, School of Pharmacy, Shahid Beheshti University of Medical Sciences, Tehran, Iran.*

## Abstract

Dibutyl phthalate (DBP) is a phthalic acid ester and is widely used in polymeric products to make them more flexible. DBP is found in almost every plastic material and is believed to be persistent in the environment. Various analytical methods have been used to measure DBP in different matrices. Considering the ubiquitous nature of DBP, the most important challenge in DBP analyses is the contamination of even analytical grade organic solvents with this compound and lack of availability of a true blank matrix to construct the calibration line. Standard addition method or using artificial matrices reduce the precision and accuracy of the results. In this study a surrogate analyte approach that is based on using deuterium labeled analyte (DBP-d4) to construct the calibration line was applied to determine DBP in hexane samples.

## Introduction

Phthalates (phthalate esters) have been used in industry as plasticizers for more than 50 years and can be found in polyvinyl chloride (PVC) and also a broad range of plastic products. Because of their high production volumes (4300000 tons per year worldwide) and widespread use, they can be detected in various environmental compartments and a constant release and diffusion into the environment is expected (1). Phthalates are known to be endocrine disrupters (2) and are believed to induce reproductive and developmental toxicity (3, 4). 

Besides being an environmental concern, the ubiquitous nature of phthalate esters becomes troublesome in the process of sample preparation in analytical procedures especially when organic solvents are to be used for extraction purposes (5, 6). Any time organic solvents are used to extract a certain analyte, because of their solubility properties, phthalate esters will also enter the solvent during the extraction process. Moreover, most of organic solvents even analytical grade solvents contain trace amount of phthalate esters which will interfere with the process of accurate quantification. If phthalates themselves are the target of quantitative analysis, finding a blank solvent becomes a real difficulty to deal with. On the other hand, the blank organic solvent when goes through the process of sample preparation, may become contaminated with unknown amounts of phthalates inadvertently. Dibutyl phthalate (DBP) , an ester of butanol and phthalic anhydride is widely used in polymers to make them more flexible (5). It shows persistence in the environment and considered to be ubiquitous (6). Various analytical methods have been applied to determine DBP in different matrices. Utilizing DBP as a plasticizer or dispersing agent in various products makes it to be easily detectable in almost all of the analytical grade solvents and materials that are commonly used for extraction and cleanup procedures in analytical methods. Thus, DBP residue migrates in to the final samples and may result in quantitative analytical measurement interferences. Considering that DBP is ubiquitous in the environment and can be found in different matrices, a true blank matrix for DBP quantification is difficult to find (7). Sorensen *et al., *(2006) measured the metabolites of phthalates in milk by liquid-liquid extraction using a liquid chromatography tandem mass spectrometry (8), in another study solid phase micro extraction combined with gas chromatography mass spectrometry was applied to determine phthalate esters in cow milk with the detection limit of 3ng/g (9).

To remove analyte residues from the blank smples, activated charcoal was applied in some of the previous studies (10). This procedure has some disadvantages such as being costly, elimination of different natural components of the matrix and finally changing the nature of matrix (11-14). Unfortunately using standard addition method to construct a calibration line also has some disadvantages like requiring a large amount of samples or being time and labor intensive which attenuate the application of this mehod (15-17).Another approach is the use of artificial matrices whenever a true blank matrix is difficult to find; In previous studies water or phosphate-buffered saline were applied as a substitute of serum or plasma (18-20). Obviously, considerable variations between artificial and real matrix can dramatically change the results (21).Providing a real blank sample is also a problem in endogenous steroids determination, Ahmadkhaniha *et al.,* (2010) performed surrogate analyte approach for the quantification of endogenous steroids in human urine which is based on isotope-labeled analyte (surrogate analyte) application instead of natural analyte to construct calibration line (22). The aim of this study is to develop a reliable and accurate method based on the application of isotope-labeled DBP (DBP-d_4_) as a surrogate analyte for DBP quantification. 

## Experimental

*Chemicals*

Dibutyl phthalate (DBP) and dibutyl phthalate-d_4_ (DBP-d_4_) were obtained from Sigma-Aldrich (Sigma-Aldrich, St. Louis, MO, USA); GC grade hexane and benzyl benzoate (BB), Ultra pure acetone and methanol were purchased from Merck (Dramstadt, Germany). BB was used as internal standard and ultra pure acetone and methanol were used to rinse all the glassware. 

*Standard solutions*

Stock solutions (1000 μg/mL) of DBP, DBP-d_4_ and BB were prepared in hexane. Calibration standard solutions containing 1, 5, 10, 25, 50 and 100 ng/mL of DBP and DBP-d_4_ with 50 ng/mL of BB were prepared daily in hexane. Determinations were performed based on the peak area of analyte to internal standard ratio. 

*Surrogate analyte method*

Sample preparation and calibration curves were constructed according to the method described by Ahmadkhaniha *et al., *(2010) for the determination of endogenous steroids in urine by surrogate analyte approach with some modifications (22). Briefly, fixed amount of internal standard solution (50 ng/mL) was added to increasing amounts of deuterium-labeled analyte (surrogate analyte) and natural analyte to construct calibration lines. Then response factor (RF) of the deuterium labeled (surrogate) analyte to the natural analyte using the most intense ion of each one to measure any isotope effect or ionization difference was calculated using the following equation:

RF =Area under the peak of deuterium labeled analyte Area under the peak of natural analyte 

If the RF value of the deuterium labeled analyte to the natural analyte is almost equal 1 , it is possible to use area under the peak of deuterium labeled analyte (surrogate analyte) to construct calibration line (22).

*Instrumentation*

GC-MS analysis was carried out using an Agilent 7000-Triple-Quad mass spectrometer coupled with 7890A gas chromatography. The column was a 5% phenyl-methyl silicone bonded-phase fused-silica capillary column (Hewlett-Packed, 30m×0.25 mm i.d., film thickness 0.25 μM). The injection port was adjusted on split less mode and the injection volume was 1 μL. The carrier gas was helium (99.999%) with 1 mL/min flow rate. The electronic beam energy was set at 70eV for mass spectra collection.

The mass detector was operated in electron impact (EI) mode using selected ion monitoring (SIM). Total run time was 21 min and the initial GC oven temperature was set at 40 ºC for 2 minutes then raised to 180 ºC at the rate of 30 ºC/min and then raised to 210 ºC at the rate of 15 ºC/min and maintained for 3 min, after that the oven temperature was raised to 250 ºC at the rate of 10 ºC/min and then to 290 ºC at 30 ºC/min and held for 4 min. 

The temperatures of mass transfer line and ion source were adjusted at 290 ºC and 230 ºC respectively. Quantifier and qualifier ions for analytes and internal standard are shown in Table 1. The fragments with m/z 149, 153 and 105 were selected as quantifier ions to monitor DBP, DBP-d_4_ and BB respectively. At the end; linearity, limit of detection (LOD), limit of quantification (LOQ), recovery and precision were assessed for method validation.

## Results and Discussion

**Figure 1:**
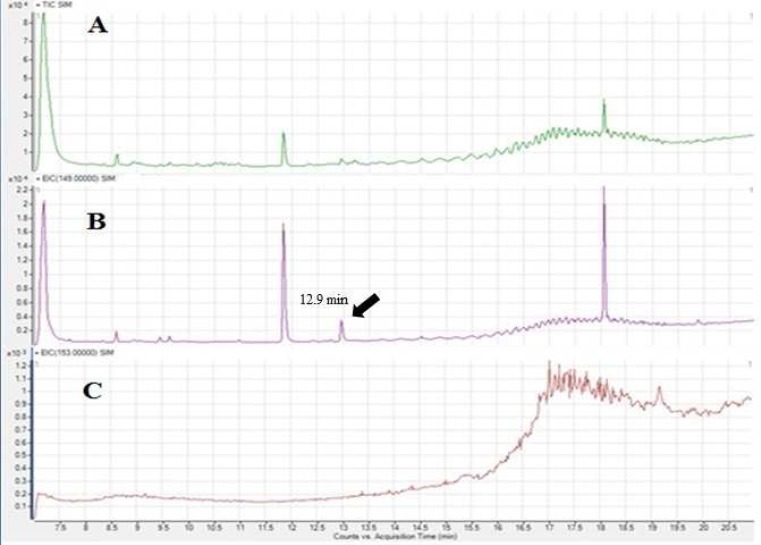
Total ion chromatogram for hexane(A), extracted ion chromatogram for m/z 149 representing DBP (B) and extracted ion chromatogram for m/z 153 representing DBP-d4 (C).

**Figure 2:**
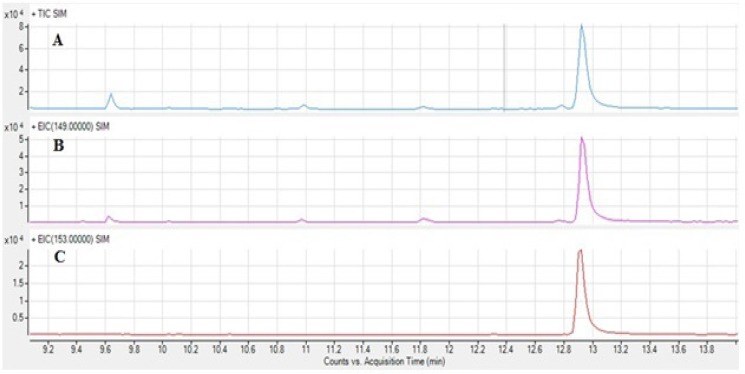
SIM GC-MS total ion chromatogram (A) and extracted ion chromatograms for DBP m/z 149 (B) and DBPd4 m/z 153 (C) in hexane sample (25 ng/ml

As mentioned before DBP is ubiquitous and can be found in different matrices and solvents. Figure 1A shows the total ion chromatogram of the hexane which was used as solvent, Figure 1B and 1C are extracted chromatograms for the m/z 149 and 153 representing DBP and DBP-d_4_ quantifier ions respectively. Although m/z 149 exists in the extracted chromatogram, m/z 153 cannot be found in the extracted chromatogram of hexane, showing that hexane sample is blank for DBP-d_4_ and hexane sample ion chromatograms (total and extracted) containing 25 ng/mL DBP and DBP-d_4_ are shown in Figure 2.

Figure 1. Total ion chromatogram for hexane(A), extracted ion chromatogram for m/z 149 representing DBP (B) and extracted ion chromatogram for m/z 153 representing DBP-d_4_ (C).

Figure 2. SIM GC-MS total ion chromatogram (A) and extracted ion chromatograms for DBP m/z 149 (B) and DBP-d_4_ m/z 153 (C) in hexane samples spiked with DBP and DBP-d_4_ at 25 ng/mL.

The next step to develop a surrogate analyte approach is the determination of response factors. The peak area of the deuterium labeled analyte to the natural analyte at equivalent concentrations were calculated and shown in Table 2. The mean response factors after 9 measurements (3 replications for 3 days) at concentrations of 1, 5, 10, 25, 50 and 100 ng/mL of DBP and DBP-d_4_ are almost equal 1 and coefficient of variations at different concentrations were less than 10% (Table 2), that permits the application of deuterium labeled analyte (DBP-d_4_) instead of natural analyte (DBP) to construct the calibration line. Quantification of DBP in hexane samples was performed using BB as internal standard, based on the peak ratio of DBP to BB. LOQ and LOD in this study were 1 ng/mL and 0.3ng/mL respectively. 

Retention time and calibration parameters of DBP are shown in Table 3.The estimated recoveries of 1, 50 and 100 ng/mL DBP spiked samples after 9 measurements (3 replications for 3 days) and the values of coefficient of variations as precision using surrogate analyte method have acceptable range and are shown in Table 4.

**Table 1:** Quantifier and qualifier ions of dibutyl phthalate (DBP), dibutyl phthalate-d_4_ (DBP-d_4_) and benzyl benzoate (BB

**Compound**	**Qualifier ions**	**Quantifier ions**
DBP	278, 223, 149	149
DBP-d_4_	282, 227, 153	153
BB	212, 194, 105	105

**Table 2:** Response factor (RF) of the DBP at different concentrations. RF = area_ deuterium labeled analyte_ / area _natural analyte_ , Standard deviation (SD), Coefficient of variation (CV%).

**Concentration (ng/mL)**	**RF (Mean)**	**Replication**	**SD**	**CV%**
1	0.962	9	0.089	9.25
5	0.958	9	0.083	8.66
10	0.971	9	0.088	9.06
25	0.991	9	0.067	6.76
50	0.983	9	0.075	7.62
100	0.986	9	0.061	6.18

**Table 3:** Retention time and calibration parameters of DBP in hexane samples. Limit of detection (LOD), Limit of quantification (LOQ

**Spiked range (ng/mL; n=18)**	**Retention time (min)**	**Regression equation**	**R** ^2^	**LOD (ng/mL)**	**LOQ (ng/mL)**
1 - 100	12.9	Y= 0.091x + 0.246	0.998	0.3	1

**Table 4:** Determined recoveries of DBP spiked samples (1, 50 and 100 ng/mL) using surrogate analyte method. Recovery (RE), Standard deviation (SD), Coefficient of variation (CV%).

**Concentration**	**Replication**	**RE% (Mean)**	**SD**	**CV%**
1 ng/mL	9	88.2	9.1	10.3
50 ng/ mL	9	93.7	6.5	6.9
100 ng/mL	9	95.6	6.8	7.1

According to the results of this study it is concluded that all of the analytical parameters of surrogate analyte approach method for DBP analyses in hexane can be appropriate and suitable and this procedure can be performed whenever a true blank matrix is unavailable or hard to find especially when determination of trace DBP level is required. Comparing to the standard addition method that is based on extrapolation and also is not quite accurate (23), constructing a calibration line using surrogate analyte approach is more reliable and feasible. 

Theoretically, the general procedure of surrogate analyte approach can be applied for DBP measurement in different matrices at trace amounts whenever a true blank matrix is inaccessible. 
